# 3D cell cultures, as a surrogate for animal models, enhance the diagnostic value of preclinical in vitro investigations by adding information on the tumour microenvironment: a comparative study of new dual-mode HDAC inhibitors

**DOI:** 10.1007/s10637-022-01280-0

**Published:** 2022-07-07

**Authors:** Sofia I. Bär, Bernhard Biersack, Rainer Schobert

**Affiliations:** Organic Chemistry Laboratory, University of Bayreuth, Universitätsstraße 30, 95447 Bayreuth, Germany

**Keywords:** Cancer research, Colon cancer, 3D cell culture, Tumour spheroids, Microtumours, HDACi

## Abstract

Anchorage-independent 3D-cultures of multicellular tumour spheroids (MCTS) and in vitro microtumours of cancer cells can provide upfront information on the effects of anticancer drug candidates, tantamount to that obtained from animal xenograft studies. Unlike 2D cancer cell cultures, 3D-models take into account the influence of the tumour microenvironment and the location dependence of drug effects and accumulation. We exemplified this by comparison of the effects of two new dual-mode anticancer agents, Troxbam and Troxham, and their monomodal congeners SAHA (suberoylanilide hydroxamic acid) and CA-4 (combretastatin A-4). We assessed the growth of MCTS of HCT116^wt^ human colon carcinoma cells exposed to these compounds, as well as the spatial distribution of dead HCT116^wt^ cells in these MCTS. Also, fluorescence imaging of live and fixed MCTS was used to assess the type of cellular death induced by test compounds. Furthermore, an innovative perfusion bioreactor system was used to grow microtumours in the presence or absence of test compounds. Both new investigational compounds led to significant reductions of the size of such MCTS and also of corresponding in vitro microtumours by inducing caspase-9 dependent apoptosis and elevated levels of reactive oxygen species. 3D multicellular tumour spheroids are easy to grow and employ for compound tests in the familiar well-plate set-up. Together with 3D microtumours grown at scaffolds in continuously perfused bioreactors they allow to study, early on in the course of drug evaluations, the communication of tumour cells with their microenvironment to an extent hitherto available only in animal experiments.

**Supplementary Information:**

The online version contains supplementary material available at 10.1007/s10637-022-01280-0.

## Introduction

Assays employing 2D monolayer cultures of adherent cancer cells are customarily used at early stages of drug development due to their little cost and high throughput screenability, in spite of their drawbacks [1]. A key disadvantage is that 2D tumour cell models disregard most of the many physiological ramifications of in vivo tumours, such as alterations in their signal transduction, gene expression, supply of nutrients and oxygen, and in their accumulation of drugs when compared with non-malignant cells and tissues [1–3]. However, the main causative factor for these shortcomings, the anchorage dependence of 2D cell cultures, can be circumvented by anchorage-independent 3D cell growth models. While cells cultured in adherent 2D systems typically react by anoikis (apoptosis induced by cell-detachment) when detached, 3D cell models behave more like real in vivo tumours [4]. They are also devoid of other intrinsic limitations of 2D monolayer cell models and thus should be given preference for preclinical screening of new investigational drugs (NID) [5]. 3D cell models are even more appropriate than 2D cell models for an early tentative dose finding, since a reduced efficacy of test compounds in 3D cell models was frequently reported, mirroring the physiological barriers of drug distribution in vivo. HCT116 colon cancer cells, for instance, were found more resistant to certain anticancer drugs when cultured in 3D *vs.* 2D cell models [6]. Therefore, 3D cell culture models are clearly preferable over the conventional 2D models, given a reasonable pricing, ease of application, and the possibility of high throughput methods.

The development of 3D cell culture applications gathered momentum in recent years [7]. Publications and patents on this topic have increased exponentially since the beginning of the twenty-first century. In the year 2021 alone, 2340 articles on “3D cell culture” were listed by the PubMed database whereas one decade before the number of articles was a mere 838. Despite their many advantages over established 2D assays, 3D models are still rarely used at early stages of drug discovery, probably due to high effort and high cost. A comparatively simple and affordable method for investigating the effects of potentially active compounds on a 3D cell culture model is presented here. In addition to analysing the growth of MCTS under the influence of test compounds, other markers commonly used in basic research on new drugs are also transferred from the 2D to the 3D cell culture model in this work.

3D cell models are a potential alternative to animal models, the indispensability of which is rather contentious. The societal pressure to do away with animal studies is building up fast, and alternatives are sought-after [8]. A current topic in cancer cell research is the development of more accurate and innovative in vitro 3D cell models. The combination of perfusion bioreactor systems with established porous chitosan-hyaluronic acid scaffolds is such an innovative approach. It allows the growth of 3D MCTS as it mimics the extracellular matrix (ECM) of a typical tumour microenvironment [9].

3D cell culture models have been described several times in recent years yet have hardly been used for the actual investigation of NID. In this study we used an anchorage independent 3D cell culture model for an assessment of the effects of two multimodal NID, Troxbam and Troxham, in comparison to the related known monomodal anticancer drugs CA-4 and SAHA (SI Fig. 1).

## Materials and methods

### Test compounds and stock solutions

Test compounds were dissolved in DMSO with a concentration of 10 mM and stored at -23 °C. Combretastatin A-4 and SAHA were purchased from TCI chemicals, Troxbam and Troxham were prepared according to literature [10].

### Cell culture

HCT116^wt^ colon carcinoma cells (DSMZ ACC 581) were cultured in DMEM (Dulbeccos Modified Eagle Medium, ThermoFisher), supplemented with 10% fetal bovine serum (Biochrom) and 1% ZellShield® (MinervaBiolabs). Unless noted otherwise, cells were maintained at 37 °C, 95% humidity and 5% CO_2_. Cells were serially passaged and only mycoplasma free cultures were used.

### MCTS

The MCTS were generated using an adapted hanging drop method [11] which was further improved for generating homogeneous MCTS. Briefly, drops containing 500 cells per 20 µL were placed on the inside of the lid of a petri dish filled with phosphate buffered saline (PBS). After an incubation period of 48 h the suspension was transferred into 1.5% agarose coated wells of a 24-well plate, transferring one drop per well in 1 mL DMEM. The spheroids thus prepared were allowed to grow for a further 5 days. This is a simple, efficient and reliable method for generating homogeneous MCTS of HCT116 colon cancer cells with a high success rate. For compound treatment 10.1 µL of 100-fold predilutions of compounds per well containing 1 mL DMEM were added 72 h before completion of the 7 d growth period. For a meaningful comparison of the 2D cell culture results and the results of the new 3D models, the MCTS were each treated with the IC_50_ (72 h) determined in 2D assays [10].

### MCTS growth quantification

The growth of MCTS was documented using an inverted transmission light microscope. The size of the MCTS was determined by measuring two orthogonal diameters (d_1_ and d_2_) per MCTS and calculating the volume using the following formula $V= \frac{4}{3}\pi \cdot r3$ with $r=\frac{(0.5*({d}_{1}+{d}_{2}))}{2}$ [11].

### Analysis of compound induced cytotoxicity

For visualising dead cells within MCTS, spheroids were stained for 30 min under cell culture conditions with 6 µg/mL propidium iodide (PI) which stains dead cells only because of their permeabilised cell membranes [12]. MCTS were then rinsed with PBS to remove excess PI. Immediately thereafter, brightfield and fluorescence images were acquired by inverted fluorescence microscopy using a Zeiss Axiovert 135 fluorescence microscope and AxioVision software. ImageJ was used for further image processing.

### Analysis of compound induced caspase 9 expression

For visualisation of caspase 9 expression in MCTS, spheroids were rinsed with PBS after 7 d of growth including a 72 h treatment with IC_50_ of test compounds [10]. They were fixed in 3.7% formaldehyde in PBS for 2 h at room temperature, followed by permeabilisation with 0.3% Triton X-100 in 1% bovine serum albumin (BSA) in PBS for a further 2 h. After rinsing with PBS, MCTS were incubated with primary antibody (Caspase 9 mouse anti-human mAb, CellSignaling, 1:300 in 1% BSA in PBS) for 16 h at 4 °C or for 2 h at 37 °C. Spheroids were rinsed once with PBS before incubation with secondary antibody (AlexaFluor 555 goat anti-mouse, ThermoFisher, 1:250 in 1% BSA in PBS) for 2 h at room temperature in the dark. After rinsing with PBS, the nuclei were stained with 1 µg/mL DAPI in PBS for 10 min at room temperature in the dark and rinsed once again, before mounting in VECTASHIELD® PLUS Antifade Mounting Medium (VektorLaboratories). Confocal fluorescence microscopy images were acquired at ex/em 555/565 nm as well as ex/em 350–380/450-460 nm and further processed using ImageJ, as well as for obtaining surface plots.

### LDH assay [13]

To measure the tendency of substances to induce necrosis, the LDH content of the media was determined which is directly linked to necrosis. After an incubation period of 7 d, 50 µL of medium supernatant of the spheroid containing wells were transferred to a 96-well plate. As a positive control, 100 µL per well of lysis solution (9% Triton-X100 in Millipore H_2_O) were added to untreated spheroids and incubated for 45 min to maintain maximum LDH release. Then 50 µL of each positive control well were also transferred into the 96-well plate. 50 µL of LDH assay buffer (223 mg of 2-*p*-iodophenyl-3-*p*-nitrophenyl-5-phenyl tetrazolium chloride, 57 mg of *N*-methylphenazonium methyl sulfate, 575 mg of *N*-adenine dinucleotide, 3.2 g of lactic acid in 480 mL 200 mM Tris–Cl, pH 8.0) were added per well. The 96-well plate was incubated in the dark for 10–30 min at room temperature. Then 50 µL of stop solution (1 M acetic acid) were added per well and the absorbance was measured at 490 nm. The mean value of background wells was subtracted from negative controls and test wells. Finally, the percentage of LDH release was calculated, setting the maximum LDH release at 100% and the negative control at 0% release. Means and SD were calculated from at least four independent experiments.

### Analysis of the compound induced ROS generation

To monitor formation of reactive oxygen species (ROS) inside the MCTS, the 2',7'-dichlorodihydrofluorescin diactetate acetyl ester (DCFH-DA), which is membrane-pervasive and per se non fluorescent, was used. After deactetylation of DCFH-DA by cellular esterases to DCFH it is oxidised by intracellular ROS to DCF which is strongly fluorescent [14]. After treatment of MCTS with IC_50_ concentrations of test compounds, DCFA-DA was added into the medium to obtain a final concentration of 20 mM, followed by an incubation for 1 h at 37 °C. The spheroids were rinsed with PBS to remove excess dye and images were immediately acquired using an inverted Zeiss Axiovert 135 fluorescence microscope with AxioVision software. For further image processing ImageJ software was used.

### Generation of in vitro microtumours

Using an innovative perfusion bioreactor system (SI Fig. 2) 3D microtumours were generated in vitro. The system enabled the growth of colon cancer microtumours whereby an exchange of oxygen and nutrients over a longer period of time was provided without having to intervene in the system. Replacing the in vivo tumour microenvironment with 3D porous scaffolds allows for the generation of reliable in vitro 3D tumour models for preclinical studies of NID [9].

In vitro microtumours were generated by means of a perfusion bioreactor system (BioMedCenter Innovations). Using Chitosan-Hyaluronic acid-based scaffolds, as established in the field of scaffold-supported 3D cell culture [9, 15, 16], the growth of colon cancer microtumours under influence of test compounds was monitored over a period of 28 d. Scaffolds were prepared according to literature [9, 15, 16]. Freeze-dried and gamma-sterilised scaffolds were placed in 24-well plates, covered with 1.5 mL DMEM (ThermoFisher), supplemented with 10% fetal bovine serum (Biochrom) and 1% ZellShield® (MinervaBiolabs), and incubated under standard cell culture conditions. The medium was replaced every 60 min, five times. Two days before seeding the scaffolds, cell pellets containing 2 10^6^ cells were generated and cultivated under cell culture conditions with regular medium change. The medium-saturated scaffolds were each seeded with a pre-incubated cell pellet and incubated overnight to ensure cell attachment. The perfusion bioreactor system was filled with 150 mL DMEM (ThermoFisher), supplemented with 10% fetal bovine serum (Biochrom) and 1% ZellShield® (MinervaBiolabs) and the seeded scaffolds were placed inside the flow chamber, followed by a perfusion period of 28 d under standard cell culture conditions. The engineered flow ensured the right nutrient supply for the growing microtumours, while the system was operated with a peristaltic pump (Masterflex L/S Digital 7551–30, Cole Parmer, flowrate 1 mL/min) providing a continuous flow, a schematic graphic representation is shown in the supporting information (SI Fig. 2). The test compounds were added via the medium at day 21 of the incubation period. After a total incubation period of 28 days the scaffolds were eventually removed and grown microtumours were measured and weighed.

### Statistics

Statistical data analysis was done using GraphPad Prism software (GraphPad Software, Inc.). Data is presented as mean ± standard error of the mean if not indicated otherwise. For determination of statistical significance one-way ANOVA coupled with Tukey's post hoc tests was used, whereas P < 0.05 were considered to indicate a statistically significant difference. Numbers of repetitions per experiment as indicated in the respective captions.

### Graphics

For creation of the artwork the following programs were used; Adobe Illustrator, ChemDraw Professional 15.0, GIMP 2.10.12, Microsoft Excel 16.0 and Power Point 16.0.

## Results and discussion

### Generation of homogeneous MCTS of HCT116 colon cancer cells

An optimal cell number for the generation of HCT116 MCTS was determined to be 500 cells. These were incubated using the hanging drop method for 2 d before being transferred to an agarose-coated 24-well plate. The agar coating of the standard 24-well plates served to avoid the need for costly low attachment plates. The growth of the MCTS was documented over a period of seven days (SI Fig. 3). During this period, a linear growth process was observed. The resulting spheroids had a diameter of > 500 µm which is considered as a critical size to closely mimic various properties of solid human tumours [17]. This model is capable to mimic tumour associated ECM interactions as well as gradients of gases, nutrients, pH and especially the delivery of potential drugs under compound treatment [1, 18].

### Influence of compounds on MCTS growth

**Fig. 1:**
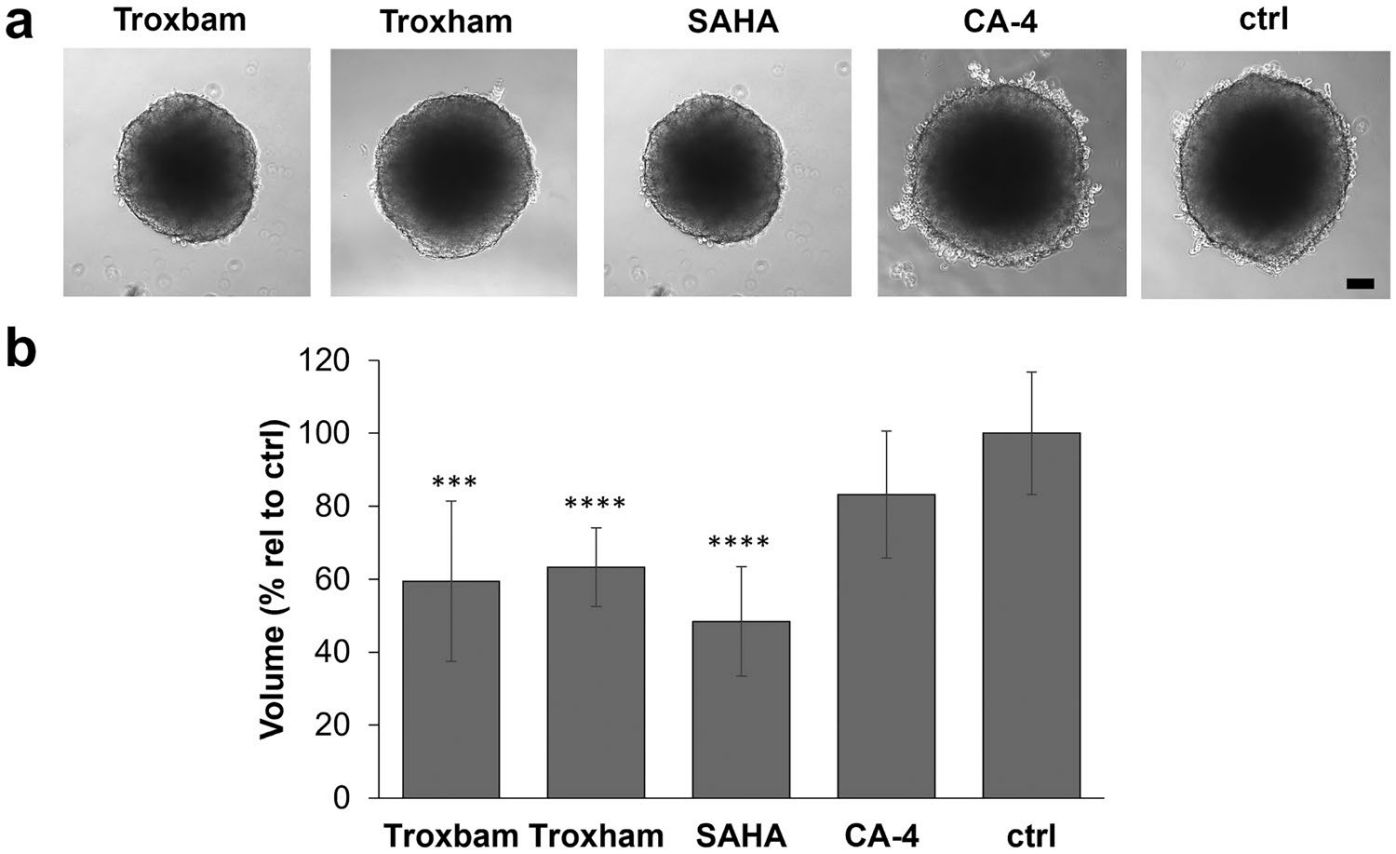
Size and volume of HCT116.^wt^ colon carcinoma MCTS after 7 d of growth with exposure to test compounds at their IC_50_ or the negative control DMSO over the last 72 h. **a** Representative images of treated MCTS, brightfield images acquired using inverted microscopy, 100-fold magnification; scale bar represents 100 µm. **b** MCTS volumes after 3 d treatment with IC_50_ of test compounds, SAHA or CA-4, related to corresponding DMSO treated controls (ctrl). Data represents the means ± SD percentage related to untreated control set to 100%. SD of n = 18, P*** < 0.001, ****P < 0.0001

First, the growth of MCTS under the influence of the test substances was observed. For treatment, the published IC_50_ values of the drug candidates Troxbam (0.9 µM) and Troxham (0.6 µM) as well as of their lead compounds SAHA (0.9 µM) and CA-4 (0.0026 µM) were used [10]. Right here, the first difference to the established 2D system became apparent. As shown in Fig. 1, unlike the other substances investigated in the 3D system, the established vascular-disrupting agent CA-4 did not lead to a significant reduction in cell growth when applied at its IC_50_ for 72 h. Despite the known higher resistance of 3D cell models to antitumoural compounds, Troxbam and Troxham reduced the growth of MCTS in the same order of magnitude as the established HDACi SAHA which indicates them as potential antitumoural drug candidates.

### Substance induced cytotoxicity in MCTS

**Fig. 2:**
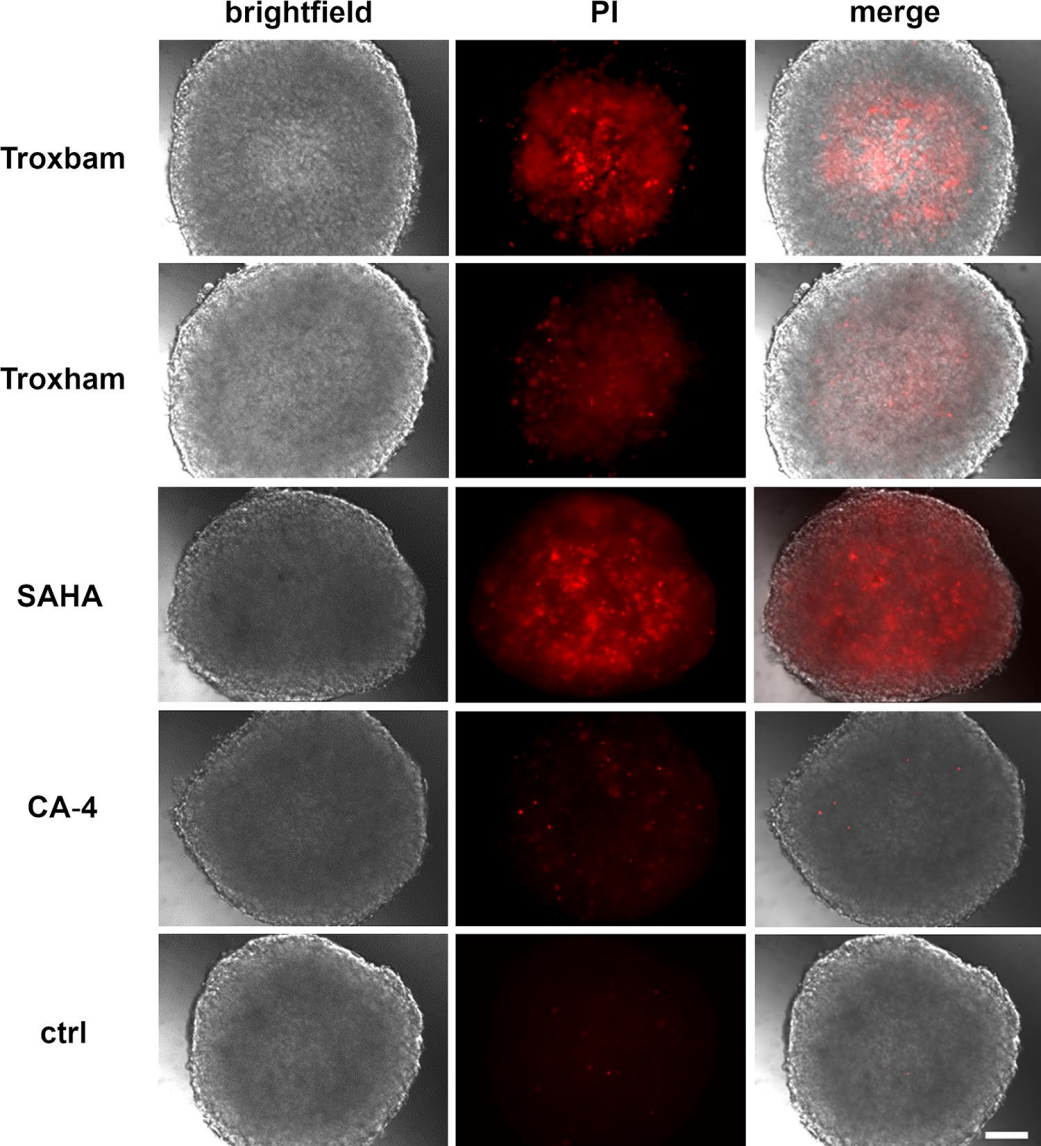
Images of PI stained MCTS, after treatment for 72 h with IC_50_ of Troxbam and Troxham, respectively SAHA and CA-4 as positive controls, and corresponding amounts of DMSO as negative control. Brightfield and fluorescence images were acquired using inverted fluorescence microscopy. The scale bar corresponds to 100 µm. The images shown are representative of three independent experiments

The cytotoxic effects of the test compounds were assessed by selectively staining the dead cells within treated MCTS with propidium iodiode (PI). As shown in Fig. 2, treatment of HCT116 MCTS with the new investigational compound Troxbam led to a distinct accumulation of red, PI-positive and thus dead cancer cells. Troxham, when applied at the same concentration range, gave a less pronounced effect, while IC_50_ of SAHA gave rise to a larger core of dead cancer cells within the respective MCTS when compared with the effect of Troxbam. In contrast, the vascular-disrupting agent CA-4 had a merely marginal cytotoxic effect on HCT116 MCTS which is in keeping with its weak growth-retarding effect as shown in Fig. 2. Besides their MCTS growth-inhibiting effect, the compounds led to a significant increase in the proportion of PI-positive and thus dead cells in the remaining spheroids, predominantly in the centre of the MCTS. This is proof that the compounds also permeate the inner regions of the spheroids. The obviously higher susceptibility of the more centreward cells is likely the result of an accumulation of metabolic products, a drop in the pH value, and a depletion of nutrients and oxygen in the core of the MCTS. A lower pH in the centre of cancer spheroids was previously reported [18].

**Fig. 3:**
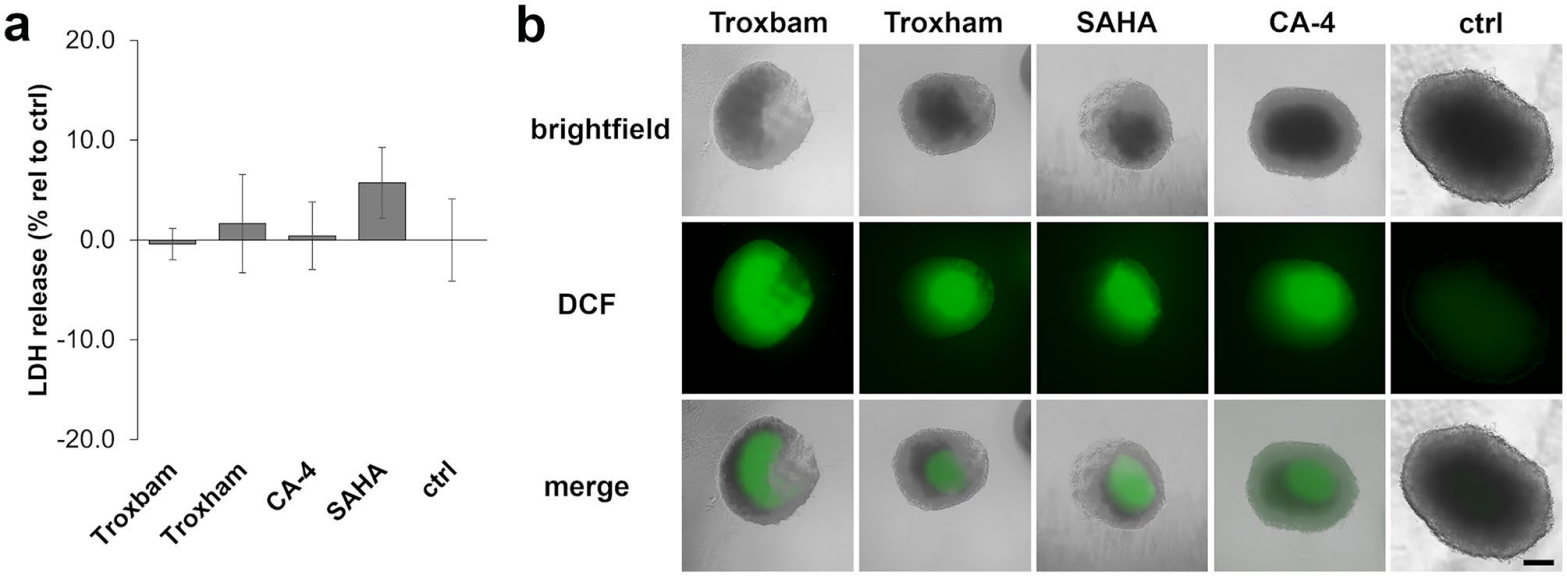
**a** LDH release by HCT116 colon cancer MCTS after treatment with IC_50_ concentrations of test compounds for 3 d. Data are presented as the LDH release relative to controls, with their basal LDH levels set to 0% and the maximum LDH release, achieved by treatment with lysis solution for 1 h, set to 100%. Data represents the mean ± SD of n = 4. **b** ROS levels in MCTS treated for 3 d with IC_50_ concentrations of test compounds, observed as DCFH fluorescence by inverted fluorescence microscopy. Images shown are representative of four independent experiments. 100-fold magnification, scale bar corresponds to 100 µm

For an assessment of a possible necrosis induction by the test compounds, the medium of the MCTS was examined for LDH after substance treatment. As shown in Fig. 3a, none of the tested compounds led to a significant enhancement of LDH levels secreted into the media. Elevated LDH release is typically associated with unselective cytotoxicity and necrosis [13]. Considering the significant growth reduction of treated MCTS and the high percentage of their PI-positive fraction, an apoptotic mechanism of cell death can arguably be assumed. A rise in reactive oxygen species is frequently observed when treating cancer cells with chemotherapeutic agents. The formation of ROS within MCTS treated with the test compounds was investigated using the membrane-pervasive pre-fluorescent DCFH-DA. All tested compounds led to a distinct enhancement of ROS levels in MCTS, with the DCFH fluorescence intensity being strongest in the middle of the spheroids (Fig. 3b). Treatment with Troxbam resulted in the highest ROS levels, yet only with a slight edge on Troxham, SAHA and CA-4. Increasing cellular ROS levels in cancer cells is considered an interesting approach to circumvent resistance and thus achieve more effective killing of cancer cells [19]. The increase in ROS levels in the MCTS is associated with the induction of apoptosis. The strong increase in ROS levels upon treatment with Troxbam, concomitant with a comparatively low caspase 9 expression, suggests caspase 8 associated apoptosis [20]. Caspase 8 associated apoptosis in conjunction with increased ROS levels in HCT116 colon cancer cells has been described previously [20].

### Substance induced expression of apoptosis associated caspase 9 in MCTS

**Fig. 4:**
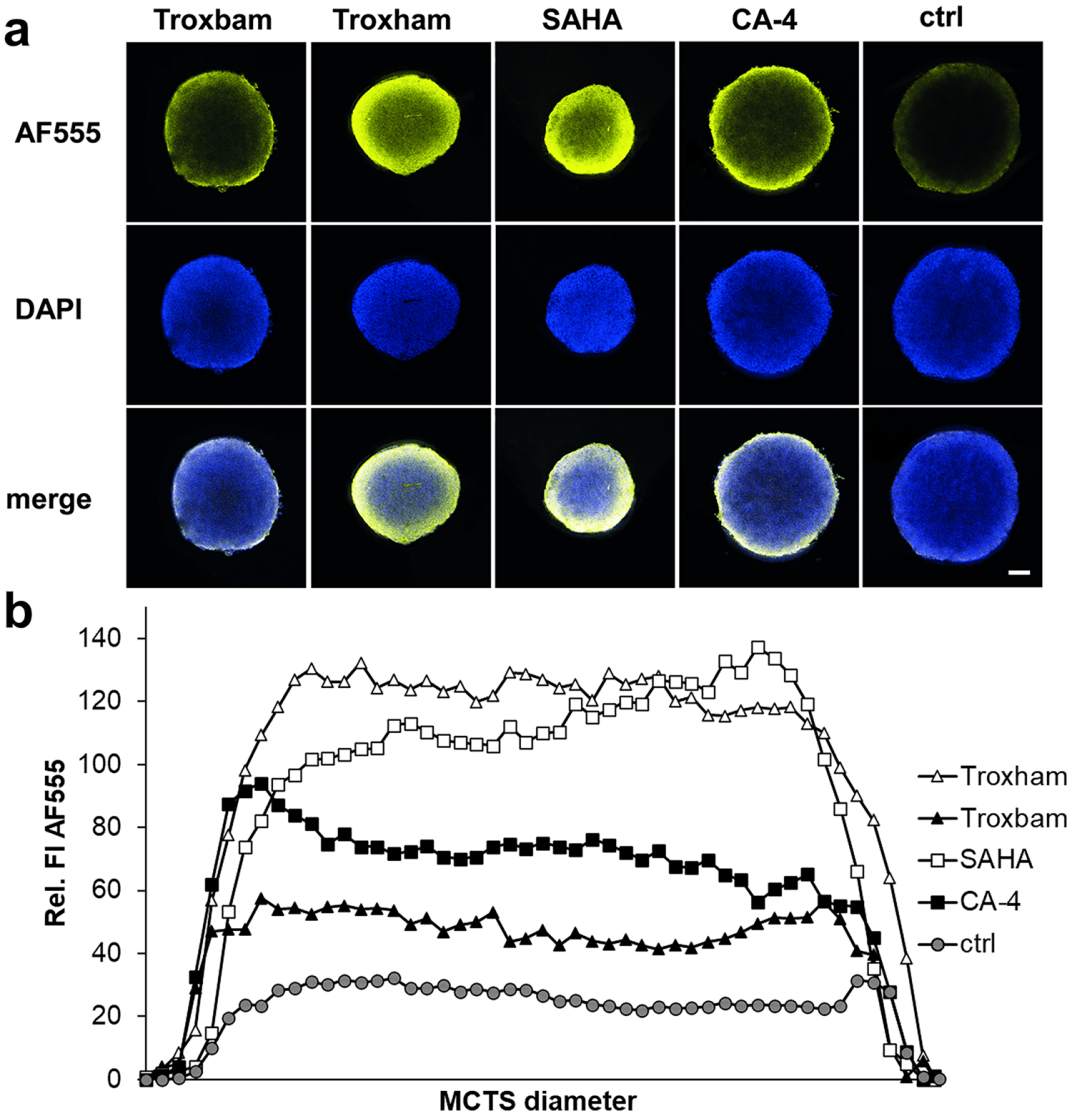
Monitoring caspase 9 expression in MCTS after substance treatment for 3 d. **a** Visualisation of caspase 9 expression in MCTS by immunofluorescence staining (AF555) and of nuclei by DAPI fluorescence staining. Images are representative of at least three independent experiments, acquired by confocal fluorescence microscopy, 100-fold magnification. Scale bar represents 100 µm. **b** Relative fluorescence intensity (FI) of AF555, representative of caspase 9 expression, plotted across the diameter of MCTS

The expression of caspase 9 upon treatment with the test compounds was investigated by immunofluorescence staining using a primary antibody for caspase 9 (mouse anti-human mAb) followed by a secondary antibody (AlexaFluor 555 goat anti-mouse). As shown in Fig. 4, an overexpression of caspase 9 was particularly pronounced in the outer rim of the treated MCTS. It also shows the average fluorescence distribution across the entire MCTS diameter for the individual test compounds. The overall strongest induction of caspase 9 expression was observed after treatment with Troxham, closely followed by SAHA. CA-4 caused only about half of this effect, while Troxbam led to a comparatively small increase in caspase 9 expression, which was, however, still twice as high as in untreated control MCTS controls. A certain basic level of caspase 9 expression is typical even of the untreated controls [21]. An overexpression of caspase 9 is a benchmark of apoptosis. When observed upon treatment with chemical compounds, elevated levels of caspase 9 expression are an indication for them being inducers of apoptosis [22]. The comparatively weak expression of caspase 9 upon treatment with Troxbam suggests the activation of a further apoptosis-inducing mechanism, considering the distinct increase in the PI-positive percentage of cells within the MCTS (*cf.* Fig. 2) and the fact that there was no increase of LDH levels (*cf.* Fig. 3a).

### Substance induced mass reduction of in vitro 3D HCT116 microtumours

**Fig. 5:**
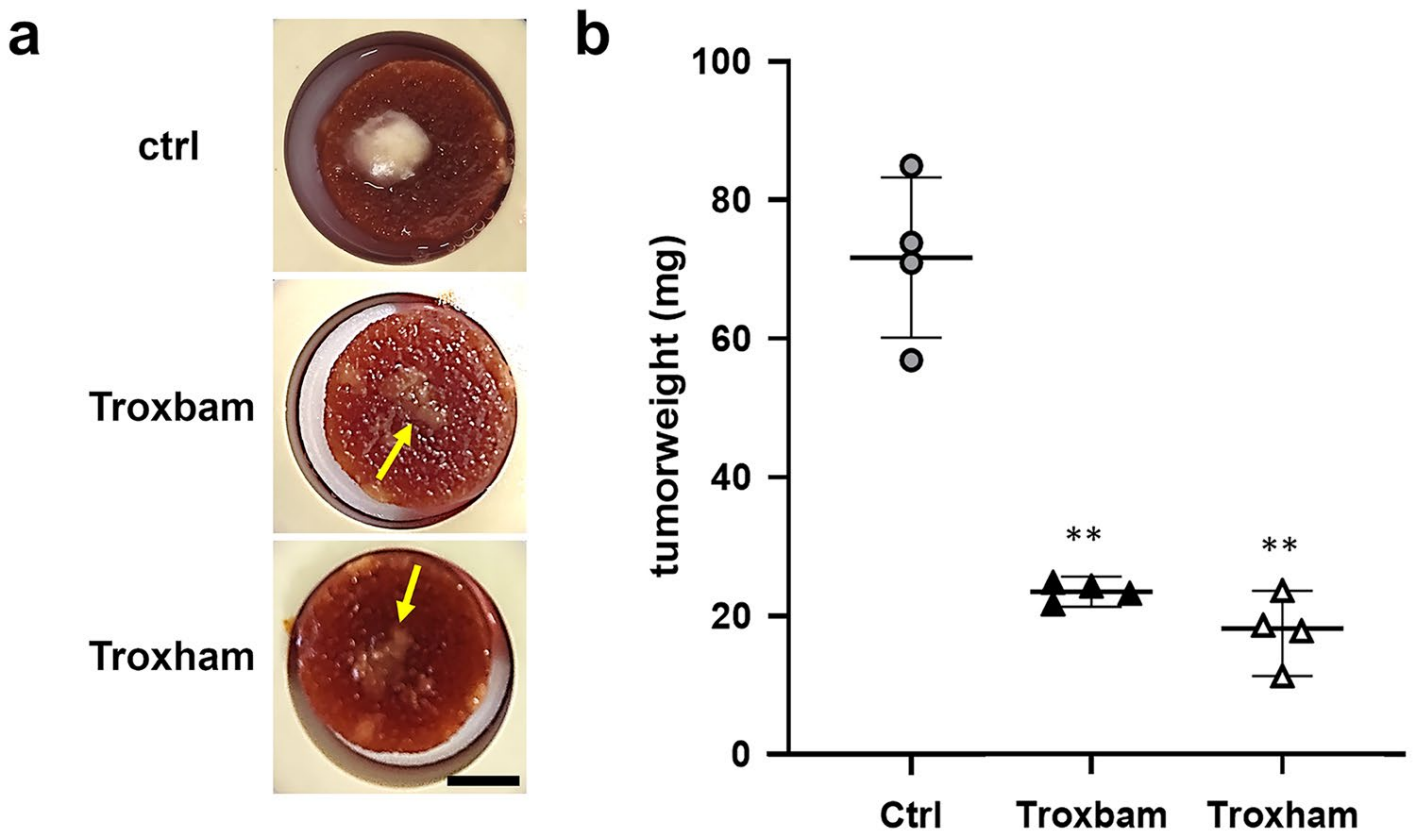
Size and weight of HCT116 colon cancer microtumours after a growth period of 28 days in a bioreactor perfusion system; substance treatment (2 IC_50_) was conducted on day 21. **a** Representative images of microtumours, grown on chitosan-hyaluronic acid scaffold. Scale bar corresponds 0.5 cm. Microtumours after substance treatment are marked with arrows. **b** Final tumour masses after total growth period of 28 days. Controls represent tumours treated with corresponding amounts of DMSO at day 21. Mean standard deviations are shown, data represent four measurements with the means ± SD. SD of n = 4, **P < 0.001

The effects of Troxbam and Troxham on the growth and persistence of 3D HCT116 microtumours were examined by means of an innovative in vitro bioreactor perfusion system. The constant perfusion of the scaffold with cell culture medium and oxygen allowed for the cells to be kept and supplied in a closed system, mimicking the conditions of in vivo studies with xenografted animals more closely than tests on MCTS in agarose-coated wells. Both new dual-mode HDAC inhibitors Troxbam and Troxham led to significant reductions of size and weight of preformed microtumours (Fig. 5).

## Conclusion

The consideration of the tumour microenvironment has been identified as an important aspect in the in vitro evaluation of new investigational anticancer drugs. While 3D multicellular tumour spheroids are easy to grow and apply for compound tests in the familiar well-plate set-up, 3D microtumours grown at scaffolds in continuously perfused bioreactors come even closer to the quality of information hitherto obtained only from in vivo experiments. In the long run, they might even replace a good deal of them, concerning the elucidation of drug induced communication of tumour cells among themselves and with the tissues they are imbedded in. 3D-based cell assays are likely the future in cancer drug screening, offering far more possibilities than the established 2D assays.

The dual-mode HDAC inhibitors Troxbam and Troxham, which we used to put the new 3D cellular assays through their paces, are interesting in their own right, even as a possible alternative to the clinically established SAHA. They led to a comparable reduction in MCTS as well as microtumour mass by inducing apoptosis in cancer cells, associated with overexpression of caspase 9 and enhanced ROS levels.

## Supplementary Information

Below is the link to the electronic supplementary material.

Supplementary file1 (PDF 261 KB)

## Data Availability

The datasets generated during and/or analysed during the current study are available from the corresponding author on reasonable request.

## Abbreviations

CA-4: Combretastatin A-4
DCFH-DA: 2´,7´-Dichlorodihydrofluorescin diactetate acetyl ester
DMEM: Dulbeccos modified eagle medium
ECM: Extracellular matrix
HCT: Human colorectal tumour
HDAC: Histone deacetylase
HDACi: HDAC inhibitor
IC_50_: Half maximum inhibitory concentration
LDH: Lactate dehydrogenase
MCTS: Multicellular tumour spheroids
NID: New investigational drugs
PI: Propidium iodide
ROS: Reactive oxygen species
SAHA: Suberoylanilide hydroxamic acid
SD: Standard deviation

## References

[1] Chaicharoenaudomrung N, Kunhorm P, Noisa P (2019). Three-dimensional cell culture systems as an in vitro platform for cancer and stem cell modeling. World J Stem Cells.

[2] Lovitt CJ, Shelper TB, Avery VM (2014). Advanced cell culture techniques for cancer drug discovery. Biology.

[3] Rodrigues J, Heinrich MA, Teixeira LM, Prakash J (2021). 3D In Vitro Model Revolution: Unveiling Tumor-Stroma Interactions. Trends Cancer.

[4] Guadamillas MC, Cerezo A, Del Pozo MA (2011). Overcoming anoikis—pathways to anchorage-independent growth in cancer. J Cell Sci.

[5] Thoma CR, Zimmermann M, Agarkova I, Kelm JM, Krek W (2014). 3D cell culture systems modeling tumor growth determinants in cancer target discovery. Adv Drug Deliv Rev.

[6] Karlsson H, Fryknäs M, Larsson R, Nygren P (2012). Loss of cancer drug activity in colon cancer HCT-116 cells during spheroid formation in a new 3-D spheroid cell culture system. Exp Cell Res.

[7] Ravi M, Paramesh V, Kaviya SR, Anuradha E, Solomon FDP (2015). 3D cell culture systems: advantages and applications. J Cell Physiol.

[8] Bédard P, Gauvin S, Ferland K, Caneparo C, Pellerin È, Chabaud S, Bolduc S (2020) Innovative human three-dimensional tissue-engineered models as an alternative to animal testing. Bioengineering 7. 10.3390/bioengineering7030115 10.3390/bioengineering7030115 PMC7552665 32957528

[9] Florczyk SJ, Wang K, Jana S, Wood DL, Sytsma SK, Sham J, Kievit FM, Zhang M (2013). Porous chitosan-hyaluronic acid scaffolds as a mimic of glioblastoma microenvironment ECM. Biomaterials.

[10] Schmitt F, Gosch LC, Dittmer A, Rothemund M, Mueller T, Schobert R, Biersack B, Volkamer A, Höpfner M (2019) Oxazole-bridged combretastatin A-4 derivatives with tethered hydroxamic acids: structure activity relations of new inhibitors of HDAC and/or tubulin function. Int J Mol Sci 20. 10.3390/ijms20020383 10.3390/ijms20020383 PMC6359144 30658435

[11] Lobjois V, Frongia C, Jozan S, Truchet I, Valette A (2009). Cell cycle and apoptotic effects of SAHA are regulated by the cellular microenvironment in HCT116 multicellular tumour spheroids. Eur J Cancer.

[12] Zhang L, Mizumoto K, Sato N, Ogawa T, Kusumoto M, Niiyama H, Tanaka M (1999). Quantitative determination of apoptotic death in cultured human pancreatic cancer cells by propidium iodide and digitonin. Cancer Lett.

[13] Chan FK, Moriwaki K. De rosa MJ (2013) Detection of necrosis by release of lactate dehydrogenase activity. Methods Mol Biol 979:65–70. 10.1007/978-1-62703-290-2_7 10.1007/978-1-62703-290-2_7 PMC3763497 23397389

[14] Rastogi RP, Singh SP, Häder D-P, Sinha RP (2010). Detection of reactive oxygen species (ROS) by the oxidant-sensing probe 2’,7’-dichlorodihydrofluorescein diacetate in the cyanobacterium Anabaena variabilis PCC 7937. Biochem Biophys Res Commun.

[15] Huang Y, Seitz D, König F, Müller PE, Jansson V, Klar RM (2019) Induction of articular chondrogenesis by chitosan/hyaluronic-acid-based biomimetic matrices using human adipose-derived stem cells. Int J Mol Sci 20. 10.3390/ijms20184487 10.3390/ijms20184487 PMC6770472 31514329

[16] Huang Y, Seitz D, Chevalier Y, Müller PE, Jansson V, Klar RM (2020). Synergistic interaction of hTGF-β3 with hBMP-6 promotes articular cartilage formation in chitosan scaffolds with hADSCs: implications for regenerative medicine. BMC Biotechnol.

[17] Nunes AS, Barros AS, Costa EC, Moreira AF, Correia IJ (2019). 3D tumor spheroids as in vitro models to mimic in vivo human solid tumors resistance to therapeutic drugs. Biotechnol Bioeng.

[18] Zagaynova EV, Druzhkova IN, Mishina NM, Ignatova NI, Dudenkova VV, Shirmanova MV (2017). Imaging of Intracellular pH in Tumor Spheroids Using Genetically Encoded Sensor SypHer2. Adv Exp Med Biol.

[19] Cui Q, Wang J-Q, Assaraf YG, Ren L, Gupta P, Wei L, Ashby CR, Yang D-H, Chen Z-S (2018). Modulating ROS to overcome multidrug resistance in cancer. Drug Resist Updat.

[20] Nishi K, Iwaihara Y, Tsunoda T, Doi K, Sakata T, Shirasawa S, Ishikura S (2017). ROS-induced cleavage of NHLRC2 by caspase-8 leads to apoptotic cell death in the HCT116 human colon cancer cell line. Cell Death Dis.

[21] Gomyo Y, Sasaki J, Branch C, Roth JA, Mukhopadhyay T (2004). 5-aza-2’-deoxycytidine upregulates caspase-9 expression cooperating with p53-induced apoptosis in human lung cancer cells. Oncogene.

[22] Druškovič M, Šuput D, Milisav I (2006). Overexpression of Caspase-9 Triggers Its Activation and Apoptosis in Vitro. Croat Med J.

