# Supplementary material for: 3D cell cultures, as a surrogate for animal models, enhance the diagnostic value of preclinical in vitro investigations by adding information on the tumour microenvironment: a comparative study of new dual-mode HDAC inhibitors
Source: Invest New Drugs. 2022 Jul 7;40(5):953–61. doi: 10.1007/s10637-022-01280-0 (PMC9395463; doi:10.1007/s10637-022-01280-0)
Supplement: Supplementary file 1 — Supplementary file1 (PDF 261 KB) [file 10637_2022_1280_MOESM1_ESM.pdf]

SUPPORTING INFORMATION

3D cell cultures, as a surrogate for animal models, enhance the diagnostic value of preclinical *in vitro* investigations by adding information on the tumour microenvironment: a comparative study of new dual-mode HDAC inhibitors

Sofia I. Bär[1,] *, Bernhard Biersack[1], and Rainer Schobert[1]

[1] Organic chemistry laboratory, University of Bayreuth, Universitätsstraße 30, D-95447 Bayreuth, Germany

* corresponding author; Sofia I. Bär, sofia.baer@uni-bayreuth.de, ORCID 0000-0002-8612-3516

Bernhard Biersack, ORCID 0000-0001-7305-346X

Rainer Schobert, ORCID 0000-0002-8413-4342

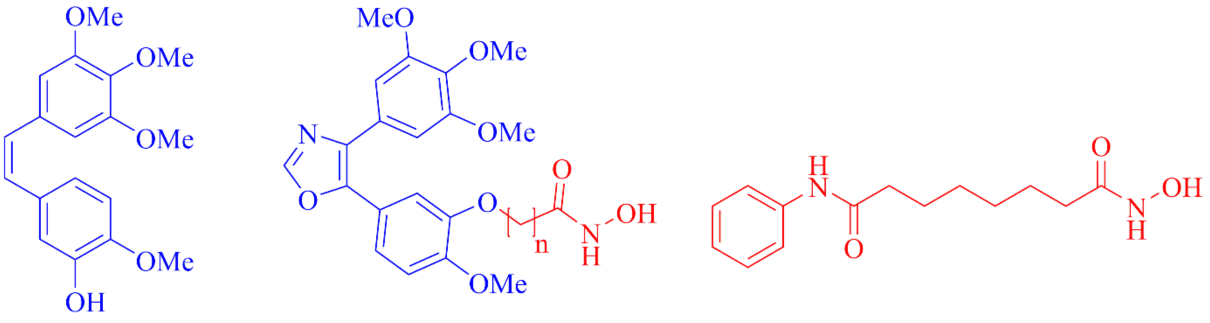

CA-4

n = 3 Troxbam
n = 5 Troxham

SAHA

SI Fig. 1 Structures of the dual-mode HDAC inhibitors Troxbam and Troxham and their known constituent congeners CA-4 and SAHA

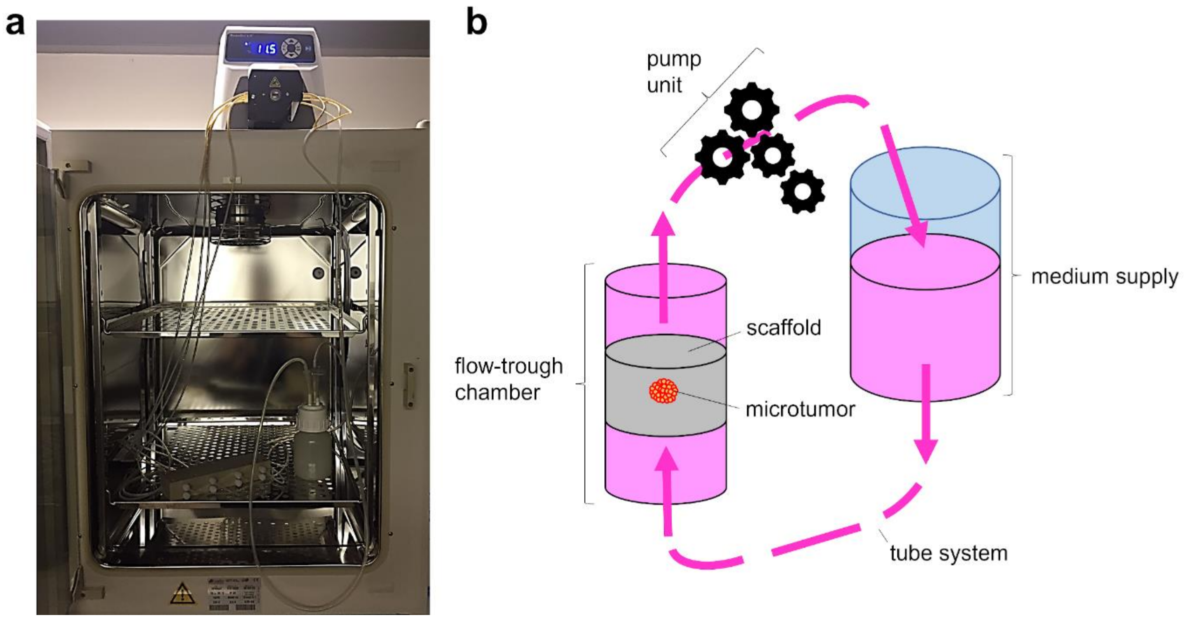

SI Fig. 2 Illustration of the bioreactor perfusion system used. a) Image of the bioreactor system, operated in a CO_2 incubator under sterile conditions. b) Schematic illustration of the perfusion bioreactor system

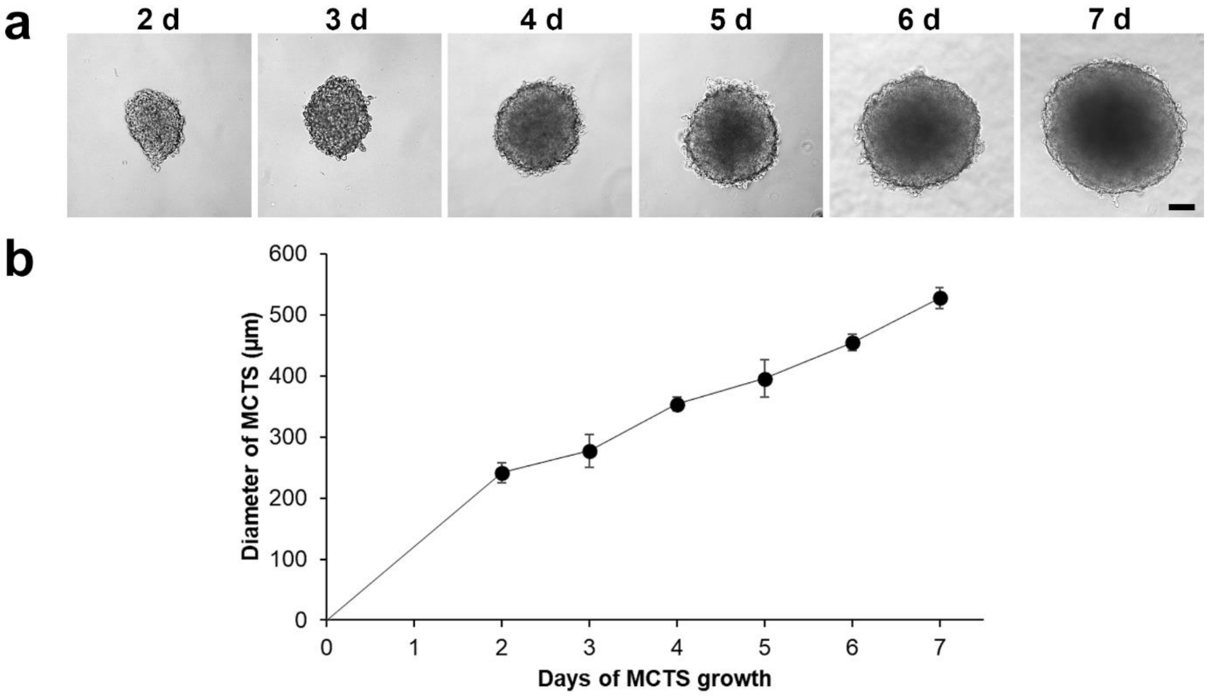

SI Fig. 3 Growth of HCT116 colon carcinoma MCTS over 7 days, measured by means of the spheroid diameter. a) Representative images of MCTS at each point of the measurement. Brightfield images were acquired using inverted microscopy. Scale bar corresponds to 100 µm. b) Graphical summary of the growth process of MCTS over a period of 7 days. Data represents mean ± SD of n = 8.
